# Multilayer perceptron-based prediction of stroke mimics in prehospital triage

**DOI:** 10.1038/s41598-022-22919-1

**Published:** 2022-10-26

**Authors:** Zheyu Zhang, Dengfeng Zhou, Jungen Zhang, Yuyun Xu, Gaoping Lin, Bo Jin, Yingchuan Liang, Yu Geng, Sheng Zhang

**Affiliations:** 1 Department of Neurology, Center for Rehabilitation Medicine, People’s Hospital of Hangzhou Medical College, Zhejiang Provincial People’s Hospital, 158# Shangtang Road, Hangzhou, 310014 China; 2 Department of Neurology, Ningbo First Hospital, Ningbo, China; 3 Key Laboratory of Bioorganic Synthesis of Zhejiang Province, College of Biotechnology and Bioengineering, Zhejiang University of Technology, Hangzhou, China; 4 Hangzhou Emergency Medical Center of Zhejiang Province, Hangzhou, Zhejiang China; 5 Department of Radiology, People’s Hospital of Hangzhou Medical College, Zhejiang Provincial People’s Hospital, Hangzhou, China; 6 College of Biotechnology and Bioengineering, Zhejiang University of Technology, Hangzhou, China

**Keywords:** Medical research, Neurology

## Abstract

The identification of stroke mimics (SMs) in patients with stroke could lead to delayed diagnosis and waste of medical resources. Multilayer perceptron (MLP) was proved to be an accurate tool for clinical applications. However, MLP haven’t been applied in patients with suspected stroke onset within 24 h. Here, we aimed to develop a MLP model to predict SM in patients. We retrospectively reviewed the data of patients with a prehospital diagnosis of suspected stroke between July 2017 and June 2021. SMs were confirmed during hospitalization. We included demographic information, clinical manifestations, medical history, and systolic and diastolic pressure on admission. First, the cohort was randomly divided into a training set (70%) and an external testing set (30%). Then, the least absolute shrinkage and selection operator (LASSO) method was used in feature selection and an MLP model was trained based on the selected items. Then, we evaluated the performance of the model using the ten-fold cross validation method. Finally, we used the external testing set to compare the MLP model with FABS scoring system (FABS) and TeleStroke Mimic Score (TM-Score) using a receiver operator characteristic (ROC) curve. In total, 402 patients were included. Of these, 82 (20.5%) were classified as SMs. During the ten-fold cross validation, the mean area under the ROC curve (AUC) of 10 training sets and 10 validation sets were 0.92 and 0.87, respectively. In the external testing set, the AUC of the MLP model was significantly higher than that of the FABS (0.855 vs. 0.715, *P* = 0.038) and TM-Score (0.855 vs. 0.646, *P* = 0.006). The MLP model had significantly better performance in predicting SMs than FABS and TM-Score.

## Introduction

Stroke is an emergency condition characterized by focal neurological deficits. Since stroke is a time-dependent disease, it is challenging to diagnose during prehospital triage stage. Approximately 25–30% of suspected stroke patients have a diagnosis of stroke mimic (SM), in which stroke-like symptoms may develop. The common SM etiologies include epilepsy, migraine, peripheral vestibular disorders, and psychiatric disorders^1,2^. Among patients with SM, some have been treated with thrombolysis therapy, leading to a waste of medical resources and financial cost^3,4^. Liberman et al.^5^ discovered that, above a threshold of 30% SM, the decision to administer thrombolysis was no longer cost-effective. In addition, although rare, it sometimes presents a risk of parenchymal hematoma^6^, symptomatic intracranial hemorrhage, and angioedema^7,8^. Therefore, efforts should be made to distinguish SM patients before stroke treatment.

Previous tools for screening SM mainly include the FABS scoring system (FABS)^9^ and the TeleStroke Mimic Score (TM-Score)^10^. FABS was a scoring system including six items: facial droop, history of atrial fibrillation, age < 50 years, systolic blood pressure < 150 mmHg at presentation, history of seizures, and isolated sensory symptoms without weakness at presentation. TM-Score was constructed by six variables, age, atrial fibrillation, hypertension, seizure, facial weakness, and NIHSS > 14. The likelihood of stroke mimic decreased as the TM-Score increases. Both scales performed well when being developed, however, there was a significant variation in the diagnostic accuracy when they were validated in external populations (sensitivity varying from 44 to 91%, and specificity of 27% to 98%)^1,9,11^.

An artificial neural network (ANN) is an artificial intelligence approach based on a large collection of neural units that models the way the brain solves problems. There are dozens of types of ANN models and multilayer perceptron (MLP) is the most commonly used and fundamental neural network structure. MLP-based models are effective in capturing nonlinear relationships that make them ideal candidates for complex and multifactorial disease classification while conventional statistical modeling, for example, logistic regression, is sometimes inadequate for establishing prediction models by comparison. In stroke area, MLP model has been applied in risk stratification of TIA and minor stroke^12^, and a recent study also proposed that MLP is ideal for prediction of disease diagnosis such as acute ischemic stroke^13^, yet the efficacy of MLP in prehospital stroke recognition has rarely been proved.

This study aimed to develop an MLP model to recognize SM in a prehospital triage stage and emergency setting and compare its efficacy with that of FABS and TM-Score.

## Material and method

### Ethical statement

This retrospective study was approved by the Human Ethics Committee of Zhejiang Provincial People’s Hospital (No. 2017KY021). All participants provided written informed consent, and the protocols were approved by the local ethics committee. All clinical investigations were conducted in accordance with the principles of the Declaration of Helsinki.

### Patient selection

We retrospectively reviewed our prospectively collected database for patients who were transferred to our hospital emergency medical service (EMS) between July 2017 and June 2021. We enrolled patients who (1) had a suspected diagnosis of stroke before admission (met any item of CG-FAST score)^14^, and (2) had a diagnosis of stroke confirmed by diffusion-weighted imaging or CT at 24 h after symptom onset. Patients were excluded if (1) clinical or imaging data were incomplete, or (2) imaging data were not available due to motion artifacts. A total of 402 patients were finally analyzed, including 82 SM patients.

We extracted data on demographics (age, gender), past medical histories (hypertension, diabetes mellitus, coronary artery disease, atrial fibrillation, previous stroke, dementia, and tumor), initial symptoms documented by the referring emergency physician (presence of “gaze deviation,” “limb weakness,” “speech problem,” “confusion,” “facial droop,” “dizziness,” “nausea and vomiting,” “inability to stand”), and admission systolic and diastolic pressure (Supplementary table I). The presence of SM was determined according to a previously published definition^2^. The patients were then randomly assigned to the training set (70%) and external testing set (30%). The dataset was randomly divided into 70% training set and 30% testing set and the data counts were 281 and 121, respectively.

### MLP modeling

All MLP models were developed using Python (version 3.8.0). The applied computational architecture was an MLP, feed-forward MLP, and back-propagation algorithm for training the feed-forward MLPs. The inputs were normalized before entering the networks using the min/max method. To train the MLP, supervised learning was performed by providing a series of input and output variables from the training dataset such that by iteratively adjusting the connection weights, a desirable input–output mapping function was generated. During the training of the model, the selection of the optimization algorithm, model structure parameters, and maximum number of iterations of model training were investigated, and the neural network architecture and connection parameters were optimized until the loss function of the model tended to be stable and the model fitting performance was the best. After appropriate training, the generalization performance of the model was evaluated using an external testing dataset.

### Feature selection and model development

The input attributes of the MLP models included clinical features extracted from the patients’ medical history that were associated with the output attributes of interest, namely age, gender, gaze deviation, limb weakness, confusion, speech problems, facial droop, dizziness, nausea and vomiting, inability to stand, hypertension, diabetes, atrial fibrillation, coronary heart disease, previous stroke, tumor, dementia, and admission systolic and diastolic pressure. In the training set, the least absolute shrinkage and selection operator (LASSO) method^15^ was used to select the most useful predictive features.

Next, in the training set, the selected features were used to develop an MLP model. Continuous variables were included as covariates and categorical variables were included as factors. These variables were included as input neurons. The activation function of each neuron was set as sigmoid function^5,16^. Adaptive momentum (Adam) algorithm was used as learning function^17^. Cross-entropy was used as loss function. Based on the training data set, the performance of the model was evaluated by ten-fold cross validation, and the optimal number of hidden layer units and the maximum iteration times of the model were determined. In order to avoid overfitting, coefficient of regularization was set as 0.001. Then, we trained the final MLP model based on the entire training dataset using the best model parameters obtained in the evaluation process.

### External testing of the MLP model and other scales for recognizing SM patients

In the study, we included the evaluation of performance of two previously published scales, namely the FABS^9^ and the TM-Score^10^. Scores of these two scales were simultaneously rated by a single independent reviewer, using clinical information electronically recorded by emergency physician and the neurologist at the time of consult. Only the clinical information available prior to treatment was used. The raters were blinded to the final diagnosis.

We then tested the FABS, TM-Score, and MLP models using the external testing set. Their predictive ability for SM was calculated and compared using the area under the receiver operating characteristic curve (AUC) in the testing set.

### Statistical analysis

Statistical analyses were conducted using SPSS (version 22.0; IBM, Armonk, NY) and Python (version 3.8.0). Continuous variables with normal distribution are presented as mean ± standard deviation, and categorical variables are expressed as percentages with corresponding 95% confidence intervals (CIs). Statistical significance was set at *P* < 0.05.

Receiver operating characteristic (ROC) analysis was performed to assess the performance of the FABS, TM-Score, and MLP models in identifying SM. The sensitivity and specificity were identified at the level that maximized the Youden value. Pairwise AUC comparisons of the ROC curves were conducted using MedCalc statistical software version 15 (MedCalc Software, Mariakerke, Belgium). Sensitivity was defined as the proportion of reference test positive (diseased) subjects who tested positive with the screening test. Specificity was defined as the proportion of reference test negative (healthy) subjects who tested negative with the screening test. Youden index integrated sensitivity and specificity information under circumstances that emphasized both sensitivity and specificity, with a value that ranged from 0 to 1. It was calculated as follows: Youden Index = sensitivity + specificity − 1. Statistical significance was set at *P* < 0.05.

## Results

### Overall characteristics

A total of 402 patients with suspected stroke were transferred to our center by emergency medical services, and 82 (20.4%) were SMs. The most frequent misdiagnoses were seizures (18%), hypoglycemia (13%), and infection (11%). The median age of the sample was 80 years (interquartile range [IQR], 69–86), and 59.7% of the patients were men. The median National Institutes of Health Stroke Scale score at presentation was 8 (IQR, 2–17). Table 1 presents the demographic characteristics of the study population.

**Table 1:** Demographic features of all patients.

Variables	n = 402
Age, y, median (IQR)	80 (69–86)
Male, n, %	240 (59.7)
**Stroke/TIA, n, %**	320 (79.6)
Hemorrhagic stroke, n, %	68 (21.2)
Ischemic stroke/TIA, n, %	252 (78.8)
LVO, n, %	119 (37.2)
Intravenous thrombolysis, n, %	72 (22.5)
Endovascular treatment, n, %	46 (14.4)

IQR, interquartile range; LVO, large vessel occlusion.

Compared with stroke patients, patients with SM were less likely to have hypertension, atrial fibrillation, previous stroke, dementia, and tumor (all *P* < 0.05). Upon initial symptoms, patients with SM were less likely to present with gaze deviation, limb weakness, speech problem, confusion, facial droop, and dizziness (all *P* < 0.05). Patients with SMs had lower admission systolic pressure (*P* = 0.021). There was no statistical difference on sex and age (Supplementary table II).

The random assignment of the training and testing sets was based on a similar proportion of SMs between the two groups (χ^2^ = 0.034, *P* = 0.854). The clinical characteristics of the patients in the training (n = 281, 67.9%) and testing (n = 121, 30.1%) sets showed no significant differences (Supplementary table III).

### Feature selection and development of the MLP model

**Figure 1:**
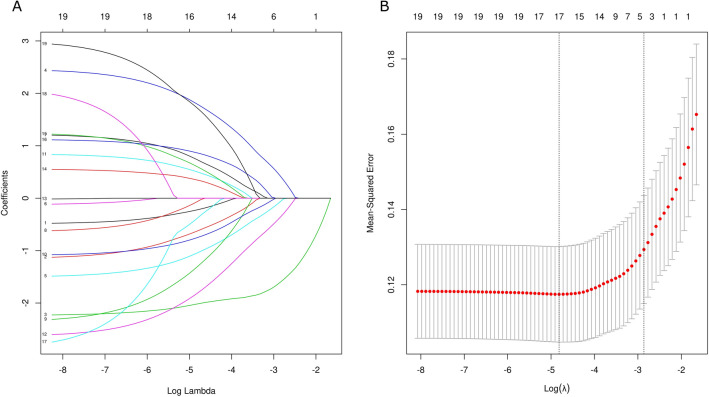
Texture feature selection using the least absolute shrinkage and selection operator (LASSO) binary logistic regression model. (**A**) LASSO coefficient profiles of the 19 texture features. (**B**) Tuning parameter (λ) selection in the LASSO model used ten-fold cross validation via minimum criteria.

**Figure 2:**
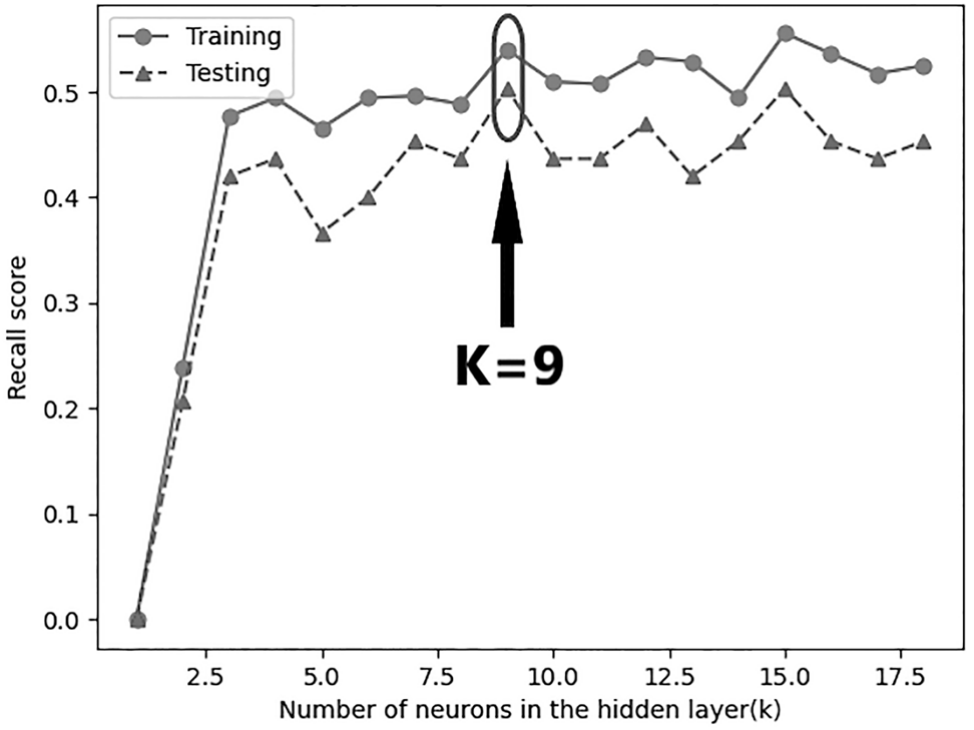
Change of recall score along with increasing numbers of hidden layer. When the number of neurons in the hidden layer reached 9, the recall score in 10 cross validation groups was the highest as the model performance remained stable.

**Figure 3:**
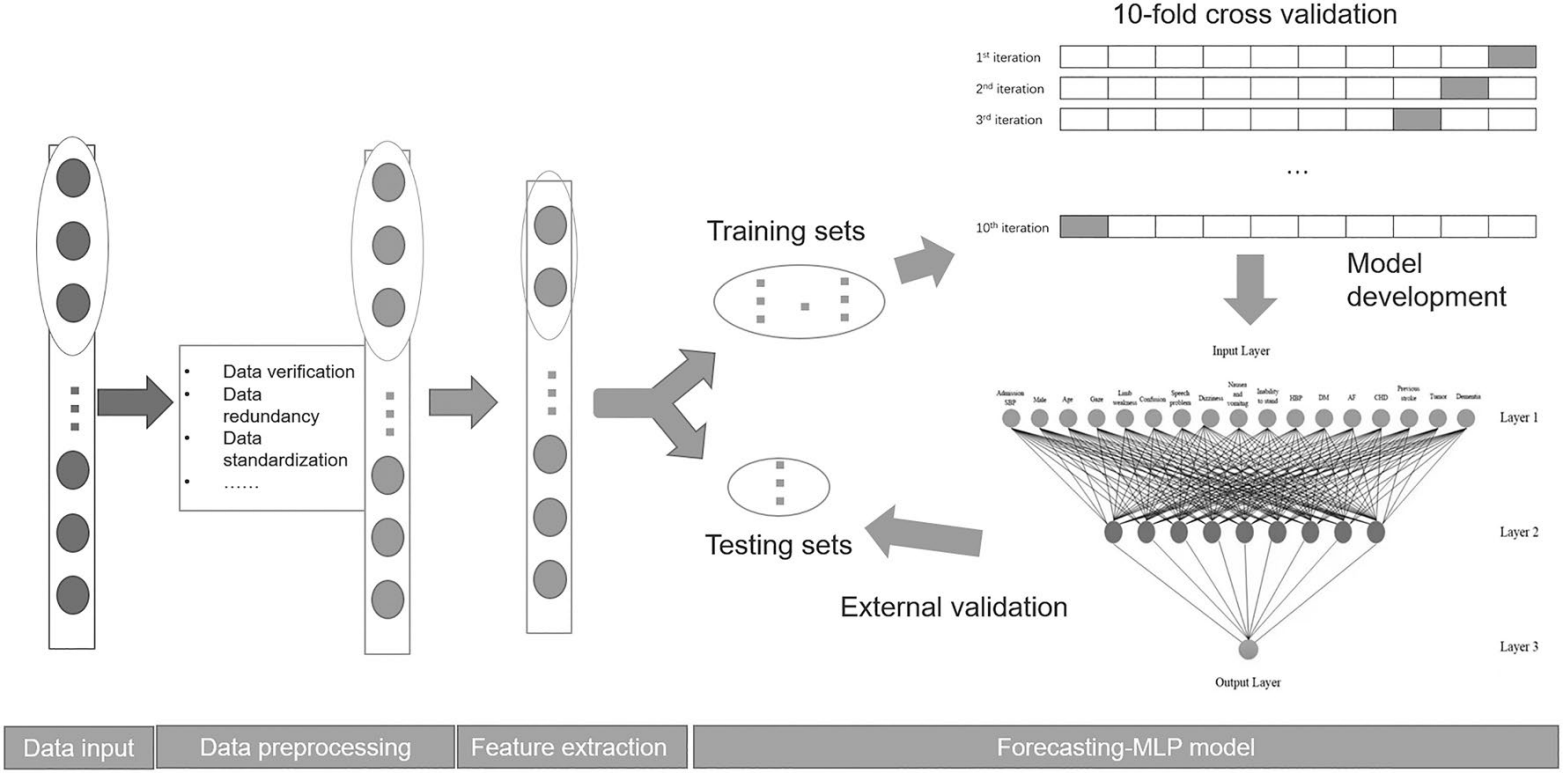
A flowchart of model development process. There are four steps in constructing an MLP model: data input, data preprocessing, feature extraction, and forecasting-MLP model.

After performing LASSO regression on the training set, 19 features were reduced to 17 potential predictors (Fig. 1). Facial droop and admission diastolic blood pressure were removed from the features. During the ten-fold cross validation process, we found that the testing errors were the lowest and reached a stable level when the number of hidden layers reached nine (Fig. 2). Therefore, there were 9 neurons in the hidden layer. Finally, we developed an MLP model using Python software (Supplementary Fig. I). To generate the MLP model, 15 categorical variables were included as covariates and two continuous variables were included. To determine the best model iteration parameters, we visualize the value of the loss function of the model iteration process based on the training data set. With the increase of iteration times, the loss function of the model drops rapidly before 1000, and it drops very slowly when the iteration times are greater than 3000. To avoid over-fitting in the training process, 3000 is selected as the parameter value of the maximum iteration times of the model. Figure 3 shows the flowchart of model development.

### MLP model performance in the training set

**Figure 4:**
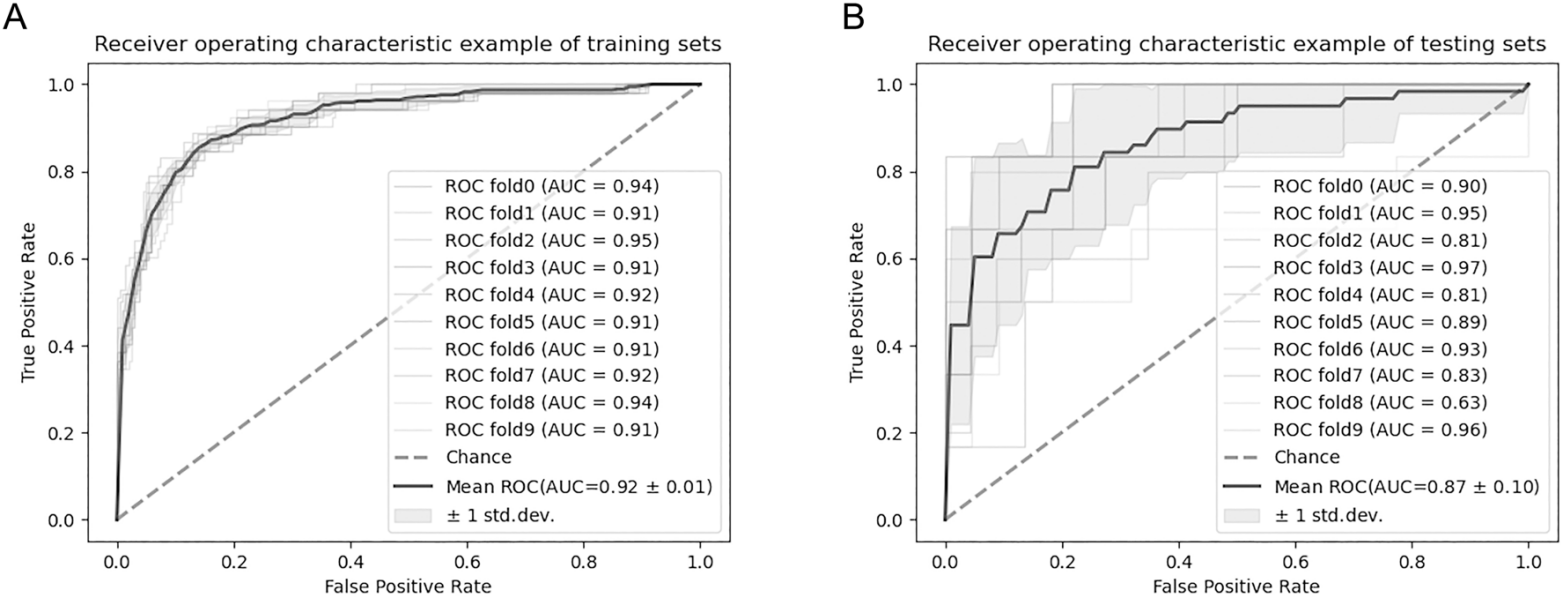
(**A**) ROC curves of 10 training sets, the mean AUC of 10 samples was 0.92 ± 0.01; (**B**) ROC curves of 10 validation sets, the mean AUC of 10 samples was 0.87 ± 0.10.

During the ten-fold cross validation, the mean AUC of MLP model in the 10 training sets was 0.92 (Fig. 4A). In the 10 validation sets, the mean AUC of the MLP model was 0.87 (Fig. 4B).

### Comparison of the SM prediction ability of MLP model, FABS, and TM-Score

**Figure 5:**
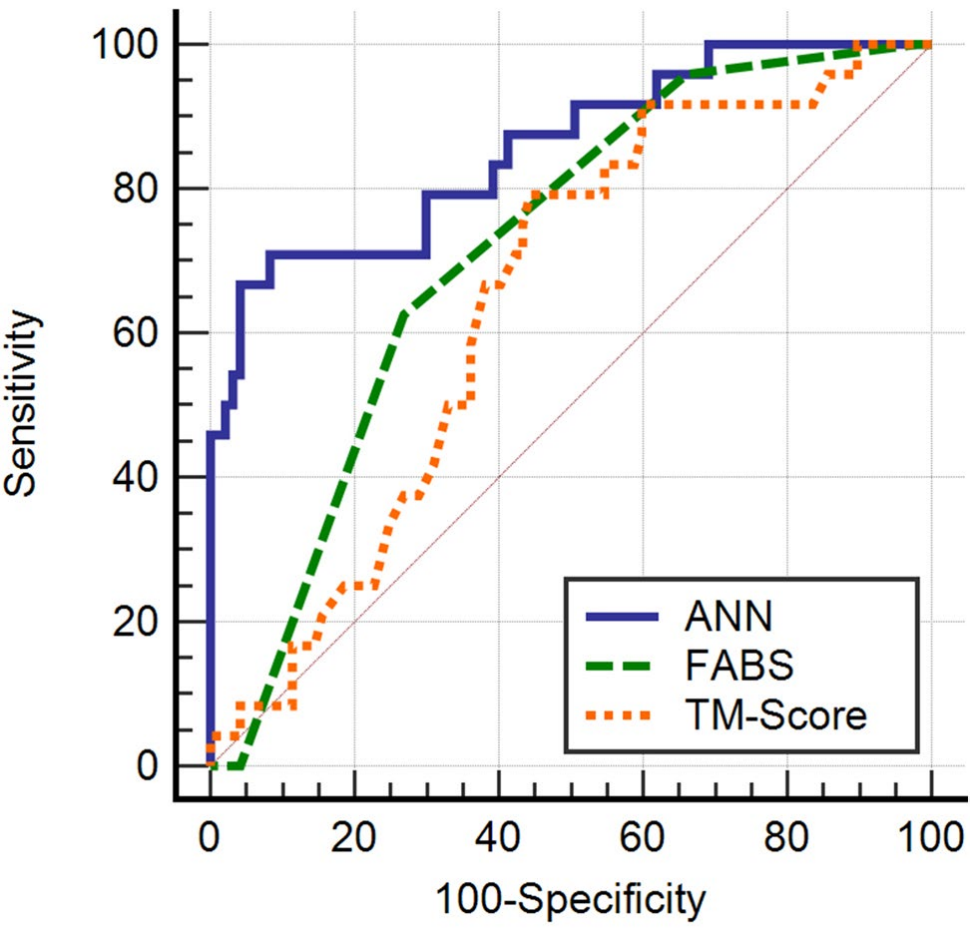
Comparison of MLP model, FABS, and TM-Score. AUC of the MLP model was significantly higher than that of FABS (0.855 vs. 0.715, *P* = 0.0038) and TM-Score (0.855 vs. 0.646, *P* = 0.006).

After the MLP model development, we used the external testing set to test the prediction ability of the MLP model, and compare it with FABS, and TM-Score. The accuracy of our MLP model in the testing set was 0.90 and its confusion matrix was shown in Supplementary table IV. In the external testing set, the AUC, sensitivity, specificity, and Youden index of the MLP model for SM diagnosis were 0.855, 0.708, 0.918, and 0.626, respectively. The AUCs of FABS and TM-Score were 0.715 and 0.646, respectively. Table 2 shows a comparison of these diagnostic parameters between the MLP model and FABS and TM-Score. Comparison of ROC analysis showed that there was no significant difference in AUCs between FABS and TM-Score (z = 1.307, *P* = 0.191), whereas the MLP model was significantly better than both FABS (z = 2.079, *P* = 0.038) and TM-Score (z = 2.736, *P* = 0.006) (Fig. 5). Overall, the MLP performance was significantly superior to both FABS and TM-Score.

**Table 2:** Comparison of the predictive performance of the MLP model and previous published scales in the testing set.

	AUC (95% CI)	Sensitivity	Specificity	Youden index
MLP model	0.855 (0.780–0.913)	0.708	0.916	0.626
FABS	0.715 (0.625–0.793)	0.625	0.730	0.357
TM-Score	0.646 (0.554–0.731)	0.792	0.557	0.384

## Discussion

In this study, we developed an MLP model to screen SM patients, and it showed the best performance in recognizing SM patients compared with FABS and TM-Score. Our study underlines the importance of applying artificial neural networks to detect SM in patients with suspected stroke.

In medical research, logistic regression (LR) methods are generally used to generate screening scales. The FABS and TM-Score used in our study were also obtained using this method. LR is a generalized linear model that processes data linearly and maps data through a sigmoid function; however, it cannot deal with linearly inseparable problems. Therefore, LR has a limited capacity for handling real-world data, as medical data usually contain complex pathological relations, especially nonlinear relationships. However, the MLP model has a good processing capacity for handling nonlinear relationships. An MLP is an architecture that learns a model from a large set of data and contains many nested layers of nodes. As input neurons accumulate, the weights on the nodes in the network are automatically adjusted to construct a model that most accurately maps inputs. In summary, the MLP model generates a nonlinear model through neurons in the hidden layer with an activation function. In recent years, MLP methods have been widely used for developing clinical prediction models, and they have been proven to exhibit better performance than conventional statistical modeling (CSM); that is, LR. Chen et al.^18^ developed an MLP model for predicting large vessel occlusion and compared it with other published scales that were developed using LR methods; they found that the MLP model was significantly better than other scales. Lin et al.^19^ used MLP models in prospectively collected stroke data and found a high accuracy in predicting functional outcome in stroke patients. Similar to the above stroke-related event, identifying SM is also a nonlinear problem; therefore, it is more reasonable to use an MLP model to predict SM. Upon developing the MLP model, the LASSO method was used to select variables. This method avoided choosing variables based on their univariable association with clinical outcomes and improved the model efficacy with lower number of variables. Therefore, facial droop and admission diastolic pressure were excluded.

MLP is better not only in methodology but also in terms of results; the ability of the MLP model to predict SM is better than that of FABS and TM-Score. A previous study showed that FABS ≥ 3 could identify patients with SM with 90% sensitivity and 91% specificity^9^. When validated externally, the AUC dropped to 0.612 with a sensitivity of 0.246 and a specificity of 0.529^1^. Similarly, in the testing set in our cohort, the performance of FABS was also poor (AUC was 0.715, with a sensitivity of 0.625 and a specificity of 0.730). Another scoring system, TM-Score, showed moderate predictive ability for SM in our cohort. The AUC of the TM-Score in the testing set was 0.646, with a sensitivity of 0.792 and specificity of 0.557. This result seems worse than that reported previously in which the AUC of TM-Score was 0.75 in the original study^10^; moreover, when validated externally, the AUC of TM-Score was still 0.75, with a sensitivity of 0.91 and a specificity of 0.59^1^. Compared with these two scoring systems, our MLP model had significantly higher accuracy. Therefore, the MLP model can be used to screen SM within 24 h of onset, and its screening ability is better than that of the TM-Score and FABS. In the future, the MLP model is expected to be applied to reduce erroneous diagnosis or misdiagnosis of stroke patients, especially for those who might be candidates for receiving thrombectomy or extended time window thrombolysis.

The items included in our MLP model and other two scales were significantly different. Firstly, both FABS and TM-Score included the evaluation of blood pressure, atrial fibrillation (AF), age, facial droop, and seizure. However, in our study, facial droop was left out after feature selection. This indicates the different model developing method between MLP and CSM, that is, the MLP model aims to integrate connections between variables to develop optimal models while CSM focus initially on the value of the variables themselves. Different methodologies result in different features of MLP model, FABS, and TM-Score. When being validated in another distinct cohort, FABS and TM-Score performed significantly different. Tu et al.^1^ validated FABS and TM-Score in a Singaporean population and found the AUC were 0.750 and 0.612, respectively. While the efficacy of MLP model is more stable when externally validated. For example, Lim et al.^20^ found the artificial neural network-based model still had excellent discrimination ability in another cohort. The phenomenon is due to the increased features and the complexity of the MLP model. In conclusion, CSM-based scales including FABS and TM-Score are less universal while machine learning-based classifiers are more promising in healthcare problems.

Notably, we found that the distribution of SM diagnoses in our cohort was different from that in previous studies. The three most common misdiagnoses of SM in our study were seizures, hypoglycemia, and infection. Seizure accounted for the highest proportion (18%), which was consistent with most previous studies that seizure was one of the three most common diagnoses of SM^6,21,22^. Seizures can also show the same gaze deviation, disturbance of consciousness, and aphasia as stroke; therefore, misdiagnosis of seizures as stroke may lead to incorrect treatment and a waste of medical resources. Except for seizures, migraine and functional disorders are the two most common SM diagnoses in previous reports. Migraine with aura usually has transient hemianopsia, unilateral movement disorder, and sensory disorder^23^. This may be confused with transient ischemic attack, leading to a misdiagnosis of cerebrovascular disease. Functional disorders were also common in other cohorts, indicating the imitative ability of stroke patients. However, in this Chinese population, we found no patients with a final diagnosis of migraine or a functional disorder, indicating a distinct distribution of SM diagnoses between other studies and ours. A possible explanation is that symptoms in patients with migraine and functional disorders usually do not last long and recover quickly. Many of these patients may refuse further examination and hospitalization, making it difficult to determine the correct diagnosis.

Our study has several limitations. First, compared with other studies, for example, with regard to FABS score, there is a selection bias in our study population because some stroke patients who were not recognized by paramedics during the pre-hospital stage were not included in the scope of our observation. Despite this, there will be a problem if we select patients according to their characteristics after arriving at the ED; that is, the symptoms of patients have changed when they arrive at the ED, which will have an impact on establishing a reliable SM recognition system that can be applied at the prehospital stage. Second, the sample size was limited, and this was a retrospective and single-center study. The algorithm should be tried on other larger population with prospective simultaneous scoring of all scores for comparison in future. Third, although the MLP model performed well in clinical use, at present, we cannot develop a concrete score system based on the MLP model because the hidden layer in the model is unexplainable. We will attempt to add procedures that can provide factor interpretation to strengthen the interpretability of the MLP model to continuously optimize the strategy of prehospital SM identification. However, it is believed that, as it is often used in medicine, the ability to explain how results are produced can be less important than the ability to produce such results and empirically verify their accuracy^24^. Additionally, CSM is based on the validation of the causal relationship; for example, smoking causes lung cancer, and intervention in smoking may affect the incidence of lung cancer. Unlike CSM, SM is a diagnosis rather than an event that needs to be analyzed through causality. Therefore, it is not essential to obtain a clear cause through the MLP model, but should focus on how to improve the diagnostic accuracy.

## Conclusions

The restricted time window requires a more accurate and intelligent classifier during prehospital triage. The MLP model is an effective tool for differentiating stroke and SM patients, and performs better than other SM probability scales for prehospital stroke recognition. Further prospective validations are needed for MLP model in a larger sample-size data.

## Supplementary Information

Supplementary Information.

## Data Availability

Datasets are available on request: The raw data supporting the conclusions of this manuscript will be made available by the corresponding author ZS, without undue reservation, to any qualified researcher.
