# Supplementary material for: Multilayer perceptron-based prediction of stroke mimics in prehospital triage
Source: Sci Rep. 2022 Oct 26;12:17994. doi: 10.1038/s41598-022-22919-1 (PMC9606292; doi:10.1038/s41598-022-22919-1)

**Supplementary table I. Independent variables included in the study**

| Variables | Data type |
| --- | --- |
| Male | categorical variable |
| Age | continuous variable |
| Hypertension | categorical variable |
| Diabetes mellitus | categorical variable |
| Coronary artery disease | categorical variable |
| Atrial fibrillation | categorical variable |
| Previous stroke | categorical variable |
| Dementia | categorical variable |
| Tumor | categorical variable |
| Gaze deviation | categorical variable |
| Limb weakness | categorical variable |
| Speech problem | categorical variable |
| Confusion | categorical variable |
| Facial droop | categorical variable |
| Dizziness | categorical variable |
| Nausea and vomiting | categorical variable |
| Inability to stand | categorical variable |
| Admission systolic pressure | continuous variable |
| Admission diastolic pressure | continuous variable |

**Supplementary table II. Comparison of baseline characteristics between patients with stroke and SMs**

|  | Stroke (n=320) | SMs (n=82) | *P* value |
| --- | --- | --- | --- |
| Male (n, %) | 197 (61.6) | 43 (52.4) | 0.133 |
| Age (IQR) | 79 (68-85) | 81 (73-87) | 0.072 |
| Past medical history (n, %) |  |  |  |
| Hypertension | 267 (83.4) | 48 (58.5) | <0.001 |
| Diabetes mellitus | 95 (29.7) | 33 (40.2) | 0.067 |
| Coronary artery disease | 54 (16.9) | 8 (9.8) | 0.111 |
| Atrial fibrillation | 105 (32.8) | 8 (9.8) | <0.001 |
| Previous stroke | 47 (14.7) | 20 (24.4) | 0.035 |
| Dementia | 18 (5.6) | 14 (17.1) | 0.001 |
| Tumor | 29 (9.1) | 15 (18.3) | 0.017 |
| Initial symptoms (n, %) |  |  |  |
| Gaze deviation | 102 (31.9) | 6 (7.3) | <0.001 |
| Limb weakness | 256 (80.0) | 25 (30.5) | <0.001 |
| Speech problem | 177 (55.3) | 24 (29.3) | <0.001 |
| Confusion | 51 (15.9) | 29 (35.4) | <0.001 |
| Facial droop | 68 (21.3) | 6 (7.3) | 0.004 |
| Dizziness | 13 (4.1) | 10 (12.2) | 0.005 |
| Nausea and vomiting | 5 (1.6) | 4 (4.9) | 0.070 |
| Inability to stand | 10 (3.1) | 2 (2.4) | 0.745 |
| Admission systolic pressure (IQR) | 161 (143-182) | 152 (136-179) | 0.021 |
| Admission diastolic pressure (IQR) | 87 (76-100) | 86 (74-100) | 0.495 |

IQR, interquartile range; SMs, stroke mimics.

**Supplementary Table III. Comparisons of features in training set (n=281) and external testing set (n=121).**

|  | All (n=402) | Training set (n=281) | External testing set (n=121) | Test value | *P* value |
| --- | --- | --- | --- | --- | --- |
| Age, y, median (IQR) | 80 (69-86) | 80 (69-86) | 79 (67-86) | Z=-0.265 | 0.791 |
| Male, n (%) | 240 (59.7) | 142 (58.4) | 76 (62.8) | χ^2^=0.695 | 0.404 |
| Admission SBP, median (IQR) | 160 (142-181) | 160 (143-183) | 160 (140-179) | Z=-0.632 | 0.527 |
| Admission DBP, median (IQR) | 87 (76-100) | 86 (76-100) | 89 (78-101) | Z=-0.811 | 0.417 |
| **Medical history, n (%)** | | | | | |
| Hypertension | 315 (78.4) | 216 (76.9) | 99 (81.8) | χ^2^=1.222 | 0.269 |
| Diabetes mellitus | 128 (31.8) | 82 (29.2) | 46 (38.0) | χ^2^=3.042 | 0.081 |
| Atrial fibrillation | 113 (28.1) | 82 (29.2) | 31 (25.6) | χ^2^=0.531 | 0.466 |
| Coronary heart disease | 62 (15.4) | 43 (15.3) | 19 (15.7) | χ^2^=0.010 | 0.919 |
| Previous stroke | 67 (16.7) | 49 (17.4) | 18 (14.9) | χ^2^=0.400 | 0.527 |
| Tumor | 44 (10.9) | 27 (9.6) | 17 (14.0) | χ^2^=1.711 | 0.191 |
| Dementia | 32 (8.0) | 22 (7.8) | 10 (8.3) | χ^2^=0.022 | 0.882 |
| **Clinical symptoms, n (%)** | | | | | |
| Gaze deviation | 108 (26.9) | 68 (24.2) | 40 (33.1) | χ^2^=3.378 | 0.066 |
| Facial droop | 73 (18.2) | 57 (20.3) | 16 (13.2) | χ^2^=2.838 | 0.092 |
| Limb weakness | 281 (69.9) | 197 (70.1) | 84 (69.4) | χ^2^=0.019 | 0.891 |
| Speech problem | 201 (50.0) | 134 (47.7) | 67 (55.4) | χ^2^=1.998 | 0.157 |
| Confusion | 80 (19.9) | 61 (21.7) | 19 (15.7) | χ^2^=1.914 | 0.167 |
| Dizziness | 23 (5.7) | 18 (6.4) | 5 (4.1) | χ^2^=0.810 | 0.368 |
| Nausea and vomiting | 9 (2.2) | 8 (2.8) | 1 (0.8) | χ^2^=1.578 | 0.209 |
| Inability to stand | 12 (3.0) | 6 (2.1) | 6 (5.0) | χ^2^=2.328 | 0.127 |
| Stroke mimic, n (%) | 82 (20.4) | 58 (20.6) | 24 (19.8) | χ^2^=0.034 | 0.854 |

IQR, interquartile range; LVO, large vessel occlusion.

**Supplementary Table IV. The MLP model prediction result on the testing set.**

| **Confusion matrix** | | Predicted condition | |
| --- | --- | --- | --- |
|  |  | Positive | Negative |
| True condition | Positive | 16 | 8 |
|  | Negative | 5 | 92 |

**Supplementary figure I. The structure of MLP model.**


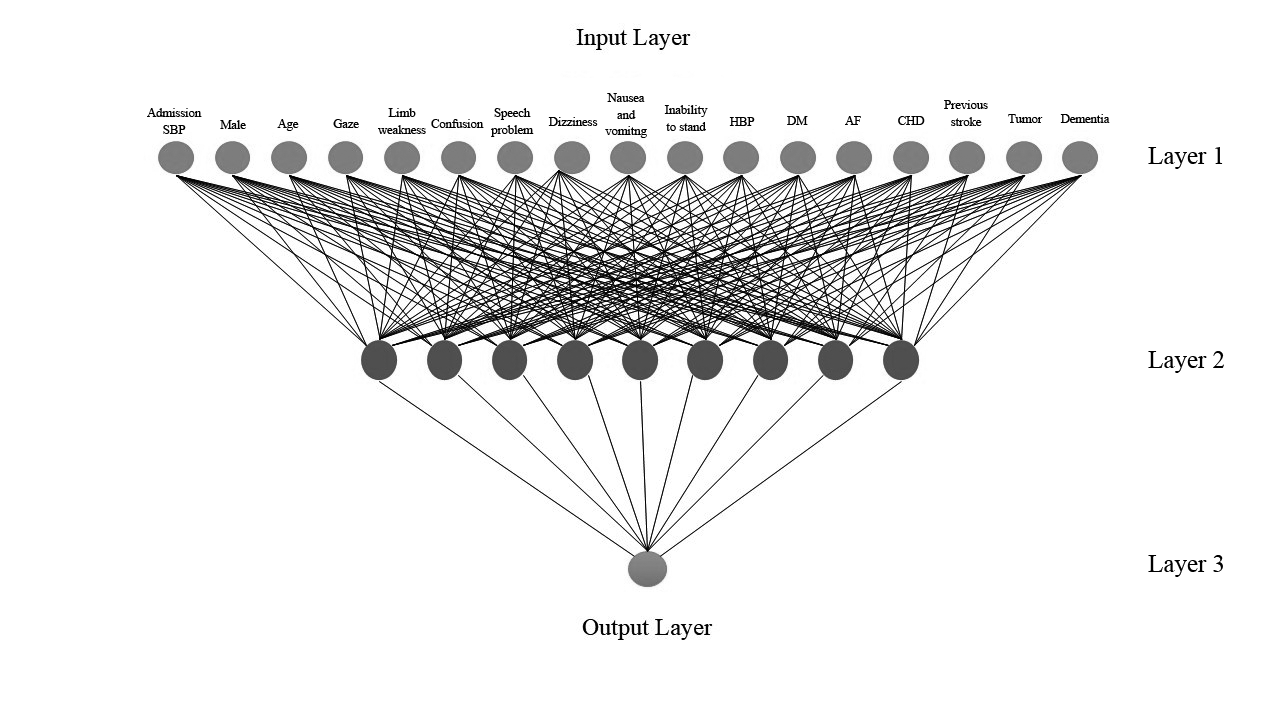

Supplement: Supplementary file 1 — Supplementary Information. [file 41598_2022_22919_MOESM1_ESM.docx]
